# ‘Waiting impulsivity’ in isolation-reared and socially-reared rats: effects of amphetamine

**DOI:** 10.1007/s00213-017-4579-8

**Published:** 2017-03-17

**Authors:** Yia-Ping Liu, Lawrence S. Wilkinson, Trevor W. Robbins

**Affiliations:** 1 Laboratory of Cognitive Neuroscience, Departments of Physiology and Psychiatry, National Defense Medical Center, Taipei, 11490 Taiwan; 2 Behavioural and Clinical Neuroscience Institute, University of Cambridge, Cambridge, CB2 3EB UK; 3 Department of Psychiatry, University of Cambridge, Cambridge, CB2 0SZ UK; 4 Behavioural Genetics Group, Schools of Psychology & Medicine, Neuroscience and Mental Health Research Institute, Cardiff University, Cardiff, CF10 3AT UK; 5 Department of Psychology, University of Cambridge, Cambridge, CB2 3EB UK

**Keywords:** Isolation rearing, Social deprivation, Impulsivity, Behavioural inhibition, Amphetamine, Dopamine, Five-choice serial reaction time task, Reward temporal discounting, ADHD

## Abstract

**Background:**

Rats reared in social isolation exhibit various cognitive and behavioural abnormalities in adulthood. However, impulsivity following this treatment still remains unclear, especially in response to medications used in attention deficit hyperactivity disorder, such as amphetamine.

**Methods:**

Using an isolation-rearing (IR) manipulation, the present study examined the effects of IR on impulsive action and impulsive choice when also treated with doses of d-amphetamine, by employing the five-choice serial reaction time task (5-CSRTT) and a temporal discounting of reward task (TDRT), respectively.

**Results:**

IR rats showed similar acquisition of the 5-CSRTT. Amphetamine increased premature responding in both groups; however, IR rats showed less responding overall. For the TDRT, IR rats revealed a greater preference for the large but delayed reward during task acquisition (i.e. were less impulsive) with a higher rate of nose poking during the delay, and exhibited a compressed dose-response function (i.e. reduced dose sensitivity) for amphetamine.

**Discussion:**

Impulsive action and impulsive choice were reduced in IR rats under certain conditions, and a blunted response to d-amphetamine was found on these measures. These reductions in impulsivity contrast with locomotor hyperactivity normally shown in IR rats and the findings have implications for the utility of IR as a model of psychopathology.

**Electronic supplementary material:**

The online version of this article (doi:10.1007/s00213-017-4579-8) contains supplementary material, which is available to authorized users.

## Introduction

Early life experience in humans can have major influences on behaviour and cognition in the adult (Bowlby 1969; Erikson 1975; Moutsiana et al. 2014). By manipulating rearing environments of rodents, for example, with the post-weaning social isolation-rearing (IR) paradigm, it has been found that deprivation of bodily contact and social interaction, including play can have profound effects on subsequent adult behaviour (Baarendse et al. 2013; Geyer et al. 1993; Hellemans et al. 2005; Sahakian et al. 1975; Zeeb et al. 2013).

Isolation rearing in rats has several effects on adult behaviour including locomotor hyperactivity and reduced prepulse inhibition which have made it a plausible model of certain psychiatric disorders including attention deficit/hyperactivity (ADHD) syndrome (see Robbins 2017 for a review). There is also considerable evidence for dopamine (DA) dysfunction in IR rats (Jones et al. 1990; Wilkinson et al. 1994; and for review, see Liu 2015 and Robbins 2017). Some of this evidence derives from apparent heightened sensitivity to the effects of amphetamine in isolation-reared (IR) rats (Sahakian et al. 1975; Wilkinson et al. 1994), which is especially relevant to the therapeutic effects of stimulant drugs in ADHD (Arnsten 2006).

In addition to hyperactivity, ADHD also entails symptoms of impulsivity (i.e. either ‘impulsive action’ or increased ‘impulsive choice’ for smaller sooner rewards compared with delayed, larger ones, i.e. during the temporal discounting of reward task, TDRT), and inattention (Sjöwall et al. 2013). Both impulsive action (as premature responses) and attention can be measured in the five-choice serial reaction time task (5-CSRTT) (Robbins 2002). Impulsive choice can also be measured in rats by the TDRT (Evenden 1999a, 1999b; Cardinal et al. 2000). A wealth of evidence links performance in both of these paradigms to dopamine-dependent functions of the nucleus accumbens (Dalley et al. 2011).

Consequently, it is pertinent to examine effects of IR, not only on measures of activity but also on impulsivity and attention using the 5-CSRTT and TDRT procedures. Previous studies have shown a tendency for IR rats to be less impulsive on some but not all measures. Dalley et al. (2002a) found that IR rats from 28 days post-weaning were in general relatively unimpaired in 5-CSRTT performance, although making the task more demanding by lengthening the inter-trial interval (ITI) produced non-significant reductions in premature responding. Zeeb et al. (2013) found similar, non-significant tendencies for premature responding in a decision-making task. Hellemans et al. (2005) likewise found no differences in performance of an analogous Go-NoGo task between IR and socially housed rats. Only Kirkpatrick et al. (2013) have found significant reduction in impulsive responding by IR rats, in a differential reinforcement-of-low-rate (DRL) paradigm. Baarendse et al. (2013) found a significant increase in premature responses in IR rats that had been re-socialized at postnatal day 43 and tested at 12 weeks of age in the 5-CSRTT, but only when the demands of the task were enhanced.

The latter study also found in contrast to no changes in the TDRT in IR rats, suggesting that their altered impulsivity did not generalize over both impulsive action and impulsive choice. However, Hellemans et al. (2005) found that IR did affect the performance of rats in TDRT, increasing preference for large rewards (reduced impulsivity). However, Kirkpatrick et al. (2013, 2014) have found consistent impairments in impulsive choice in IR rats. Consequently, there is an evident need for a definitive study that compares effects of IR on measures of both impulsive action and impulsive choice.

There is a similar need for a detailed dose-response study of the effects of amphetamine on behaviour in both test paradigms. Dalley et al. (2002a) found that IR rats exhibited less of the usual increase in premature responses at a single dose of d-amphetamine. In contrast, Zeeb et al. (2013) found that the amphetamine-induced increase was similar for socially reared and IR rats. Baarendse et al. (2013) investigated the effects of only a single dose of d-amphetamine for both 5-CSRTT and impulsive choice, also finding no differences between IR and social groups. Kirkpatrick et al. (2013, 2014) have not investigated effects of amphetamine with IR using their paradigms.

Consequently, we compared effects of IR (though without the additional complicating effects of re-socialization, as in Baarendse et al. 2013 or environmental impoverishment, as in Kirkpatrick et al. 2013, 2014) in two main measures of impulsive behaviour, the 5-CSRTT and the TDRT, as well as the dose-effects of amphetamine, while also including measures of locomotor activity, to provide the first comparison of these three measures following IR in the same study.

## Materials and methods

### Animals

After weaning, Lister Hooded male rats were received from the supplier (Harlan Olac, Bicester, UK) on postnatal days 21–23. Upon receipt, animals were housed in a temperature and humidity-controlled holding facility (21 ± 1 °C) on a 12-h light/dark cycle (lights on at 07:00, at 100 lx). Rats in the IR group were housed in single cages (cage size 43 × 25 × 20 cm) although they could see, hear and smell other rats in the colony. The ‘social rearing’ (SR) group derived from the same batch of animals as the IR animals were housed in pairs in home cages of equal size to the IR group. Separate groups were used for 5-CSRTT (12 IR, 12 SR, experiment 1) and TDRT (12 IR, 12 SR, experiment 2). For timeline indicating the age of rats in different manipulations across the experiments, see supplement Fig. 1. The animals were food restricted to 20 g/rat/day of rodent chow so that they reached 90% of free-feeding weight. Testing occurred between 0800 and 1800 with individual animals tested at the same time each day. All animals used in the studies were treated in accordance with the U.K. Animals (Scientific Procedures) Act 1986.

### Validation of the effects of isolation rearing

Before carrying out the behavioural experiments, it was important to show that the IR procedure provided comparable effects to those previously published. We validated the procedure by testing locomotor activity in each group of animals before commencing 5-CSRTT or TDRT testing. Testing was conducted between 0800 and 1600 hours, coinciding with the dark period. Rats were placed individually in photobeam activity cages, which had two parallel horizontal infrared beams positioned across the long axis of the cage 1 cm above the floor, for 2 h. The cages were controlled and the data were collected at 5-min intervals using a BBC microcomputer programmed in BBC Basic. Interruption of either beam resulted in an incremental count for that cage. Both total beam breaks and consecutive beam breaks (‘runs’) were collected. Runs required the subject to break the infrared beam at each end of the cage in sequence, providing an estimate of locomotion as opposed to general activity.

### Experiment 1: impulsive action: 5-CSRTT

#### 5-CSRTT apparatus

A standard 5-CSRTT apparatus (Paul Fray Ltd., Cambridge, UK) was used as others (Carli et al. 1983; Cole and Robbins 1989), consisting of an aluminium operant chamber (25 × 25 cm) with a food magazine at the front and five apertures in the curved rear wall; the magazine and apertures were illuminated with 3-W bulbs and could be monitored with infrared photocell beams. A 3-W house-light illuminated the chamber and the whole apparatus was contained within a sound-attenuating wooden box. Fans provided ventilation and helped to mask background noise. On-line control of the apparatus and data collection was performed using a microcomputer [Acorn Archimedes 440/1 programmed using BBC language (Paul Fray Ltd., Cambridge, UK)].

#### Behavioural training procedure

5-CSRTT training procedures from this laboratory have already been described (Bari et al. 2008). Briefly, at approximately 12 weeks of age, the animals were first habituated to the chambers over 2 days and the magazine was baited with pellets so they learned that food was available during 20-min sessions. Next, the rats were moved onto a fixed interval schedule. For 3 days, pellets were delivered every 20 s to the magazine during a 20-min session. Then, from day 6 of training, rats could initiate a trial by making a nose poke to the magazine, using a simple reaction time task (SRTT) with 40 min sessions or 100 trials, whichever came first. At the beginning of these sessions, the house-light was illuminated and a food pellet was delivered to the magazine. When the animal retrieved this pellet, the first trial was initiated, and after a delay, the stimulus light was presented over the central hole (stimulus duration gradually reducing from 60 to 2 s). If the rat then broke a photobeam crossing by nose poke, a food pellet was delivered into the food tray. An inter-trial interval (ITI) began when the food pellet was retrieved, with the next stimulus light presented after the ITI. During the limited hold period (5 s), if the rat failed to nose-poke the stimulus hole (omission), nose-poked the wrong hole (incorrect response) or nose-poked before the stimulus was presented (premature response), a 5-s period of darkness would occur, during which no stimuli were presented. Then, the animal could start a new ITI by a nose-poke to the magazine. When rats reached criterion on this task (omission <20%, accuracy >80%, premature responses <30), the same procedure was used, but any of the 5 holes illuminated in a pseudo-random manner, introducing the 5-CSRTT. The stimulus duration was gradually decreased to 0.5 s as animals progressed to each stepped duration (2, 1.5, 1, 0.75, and finally 0.5 s) according to criteria (omission <20%, accuracy >80%, premature responses <30) and when they had reached this criteria on the final stage on 3 continuous sessions, training was completed.

#### Variable ITIs: temporal unpredictability (17–18 weeks of age)

Once the animals had acquired the task, manipulations were introduced to challenge their performance under conditions of temporal unpredictability of the visual target stimuli using sets of both short (2, 3, 4, 5 s) and long ITIs (5, 6, 7, 8 s). The animals were run on variable ITI sessions, where equal numbers of the ITI durations (i.e. 25 trials) were randomly distributed across the session. This experiment aimed to investigate if the unpredictable stimulus presentation would affect performance on the task.

#### d-Amphetamine treatment

After the behavioural challenges, we investigated the effects of d-amphetamine on 5-CSRTT performance under both standard testing condition (5 s ITI) (at 18–19 weeks of age) and long variable ITIs (5, 6, 7, 8 s) (when the rats were 19–21 weeks old). Rats received a sequence of IP injections of vehicle (0.9% saline solution) or d-amphetamine sulphate (dissolved in the same vehicle) and administered in a volume of 0.1 ml/100 g body weight, 15 min prior to testing. The doses used were 0.4, 0.6 and 0.8 mg/kg, and the injection order of doses was based on a Latin Square design. Each drug test session was preceded by two control sessions, when saline vehicle was administered and baseline performance re-established.

#### Data analysis

Accuracy was calculated as the percentage of correct responses. Premature responses were recorded if the rat responded before the stimulus presentation. Perseverative responses occurred when the rat responded additionally following a correct response and prior to collecting the reward. Latency to a correct response was the amount of time elapsing between the onset of the stimulus and the rat’s correct nose-poke response. Sessions to criteria was a measure of acquisition, the number of sessions that the rats needed to acquire each sub-stage of the SRTT and 5-CSRTT training.

#### Statistical analysis

For each variable, ANOVA was used with one between-subject (rearing condition), and one or two repeated factors (amphetamine dose and variable ITI length). Following significant main effects or interactions, subsequent post hoc comparisons using Dunnett’s test were carried out where appropriate. For both IR and SR groups, the results were expressed as means. The criterion for statistical significance was *p* < 0.05. The standard error of mean (SEM) was used for an index of variation. The standard error of the differences between means (SED) was taken from the interaction component of the ANOVA and could be used as the denominator for post hoc comparisons with Student’s *t* test (Cochran and Cox 1957). It can be used for visual evaluation for the difference between two mean values (Cardinal et al. 2000).

### Experiment 2: impulsive choice: temporal discounting of reward task

#### Apparatus

Four standard operant chambers were used for this experiment (30 × 24 × 30 cm; Modular Test Chamber ENV-007, Med Instruments Inc., Georgia, Vt., USA). Each chamber was fitted with a 2.8-W overhead house-light and two retractable levers with a 2.8-W stimulus light above each lever. Between the two levers was a magazine where pellets could be delivered, with a light and photobeam to detect nose pokes. The chambers were enclosed within wooden sound-attenuating boxes fitted with fans. The apparatus was controlled by software written by R. N. Cardinal in Arachnid (Paul Fray Ltd., Cambridge), running on an Acorn Archimedes series computer.

#### Behavioural training procedure

After habituation, at about 12 weeks of age, rats were trained on a fixed-ratio 1 schedule for 3 days, where a lever press resulted in the delivery of a food pellet. The animals were trained to a criterion of 50 presses in 30 min for both the left and then the right levers. Then, the rats were trained on a simplified version of the TDRT. The session began with the levers retracted and the lights extinguished. Every 40 s, a trial would begin with illumination of the house-light and the tray-light. The rat was required to make a nose-poke response within 10 s, or the chamber returned to darkness. If the subject made a nose-poke within the time limit, the tray-light was extinguished and a single lever was presented (in every pair of trials the left and right levers were both presented once, the order within the pair was random). If the rat failed to press the lever within 10 s, the lever was retracted and the lights extinguished, but if the rat responded, the house-light was switched off and a single pellet was delivered immediately. The tray-light was illuminated until the rats collected the pellet or a 10-s collection time limit elapsed. This taught the animals to initiate trials and respond to the levers. When the rats completed 50 successful presses out of the 60 possible trials during the training session, the fixed trial length was increased from 40 to 70 s, and then in a step-wise fashion up to 100 s. There was no delay during training. When the rats achieved the criterion on the simplified task, they were trained on the final schedule, where the delay was introduced.

The delay trials began every 100 s, with the house and tray lights illuminated, but the levers retracted. The rat was required to make a nose-poke to the magazine, ensuring it was located centrally. If the rat did not respond within 10 s of the start of the trial, the lights extinguished until the next trial began, and it was scored as an omission. If the rat nose-poked, the tray-light extinguished and one or both levers were presented, depending on if it was a forced-choice or free-choice trial. One lever was designated the ‘delayed’ lever, the other the ‘immediate’ lever in a counterbalanced left/right order. If the rat did not respond within 10 s of the lever presentation, the chamber was reset to the inter-trial state until the next trial began and the trial was recorded as an omission. When a lever was chosen, both levers were withdrawn and a pellet was delivered immediately to the illuminated food magazine, or 4 pellets were delivered after a delay, dependent on whether the choice was made on the ‘immediate’ or ‘delayed’ lever. Multiple pellets were delivered 0.5 s apart and the time from the delivery of the first pellet until a nose-poke occurred was recorded as the collection latency. If the rat did not collect the food within 10 s of its delivery, the magazine light would be switched off and the chamber returned to the inter-trial state.

Each session consisted of five blocks with systematically varied delays across the session. Each block began with two forced-choice trials followed by ten free-choice trials. For forced-choice trials only one lever was presented so there was no ‘choice’ (one trial for each lever, in randomized order), whereas on free-choice trials, both levers were presented. These ‘forced-choice’ trials were designed to ensure that rats were informed about the given delay at the start of each block and had the opportunity to sample both the small and the large reinforcer. Delays for each block were 0, 10, 20, 40 and 60 s, respectively. As trials began every 100 s, the total length of a session was 100 min and rats were tested for one session daily.

#### Behavioural challenges

When the animals reached standard performance on the task, two manipulations were introduced, each after at least four training sessions under standard parameters. First, the delays were removed from the task (i.e. all delays were set to zero, to test the effects of reinforcer magnitude per se). Second, when rats chose the delay lever, the reward was changed to 2 pellets (i.e. reinforcing ratio was changed from 4:1 to 2:1).

#### d-Amphetamine treatment (19–21 weeks of age)

After the behavioural challenges, we investigated the effects of d-amphetamine on TDRT performance. Rats received a sequence of intraperitoneal (IP) injections of vehicle (0.9% saline solution) or d-amphetamine sulphate (dissolved in the same vehicle) and administered in a volume of 0.1 ml/100 g body weight, 15 min prior to the TDRT session. The doses used were 0.4, 0.8 and 1.5 mg/kg (this use of the higher dose of 1.5 mg/kg examined whether a dose greater than 0.8 mg/kg would expand or lessen the drug-induced group difference observed in experiment 1)**.** The order of doses was based on a Latin Square design and allowed at least 2 washout days between treatments.

#### Data analysis

The measures of interest for this study were the percentage of trials of rats to choose the large but delayed reward (our behavioural index of choice impulsivity) and the number of nose pokes during the delay.

#### Statistical analysis

The method of statistical analysis used in experiment 2 was identical to that used in experiment 1.

## Results

### Locomotor activity

For locomotor activity, IR rats were significantly hyperactive in comparison to SR rats with a main effect of rearing condition in both experiment 1 [breaks 2498 ± 134 for SR and 3580 ± 151 for IR, *F*(1,22) = 4.69, *p* < 0.05; runs 635 ± 47 for SR and 883 ± 53 for IR, *F*(1,22) = 4.84, *p* < 0.05] and experiment 2 [for breaks 2141 ± 158 for SR and 2983 ± 172 for IR, *F*(1,22) = 8.13, *p* < 0.01; for runs 587 ± 61 for SR and 736 ± 83 for IR, *F*(1,22) = 5.48, *p* < 0.05].

### Experiment 1: impulsive action

#### 5-CSRTT acquisition (Fig. 1)

Rearing condition had no effects on acquisition sessions to reach the criteria of task performance for 5-CSRTT in percent correct, correct latency and premature responses [percent correct, *F*(1,22) = 0.07; correct latency, *F*(1,22) = 0.05; premature responses, *F*(1,22) = 0.08; ns for all], indicating that SR and IR rats were equal in their abilities to learn the task. However, IR rats exhibited a tendency towards an increase in perseverative nose pokes during the acquisition stage [*F*(1,22) = 1.98, *p* < 0.25 for the one choice task and *F*(1,22) = 2.09, *p* < 0.25 for the five-choice task]. For both groups, there was a general increase in correct latency with increasing laterality of response location [main effect of stimulus position [*F*(4,88) = 5.15, *p* < 0.01 for SR rats and [*F*(4,88) = 5.42, *p* < 0.01 for IR rats].

**Fig. 1:**
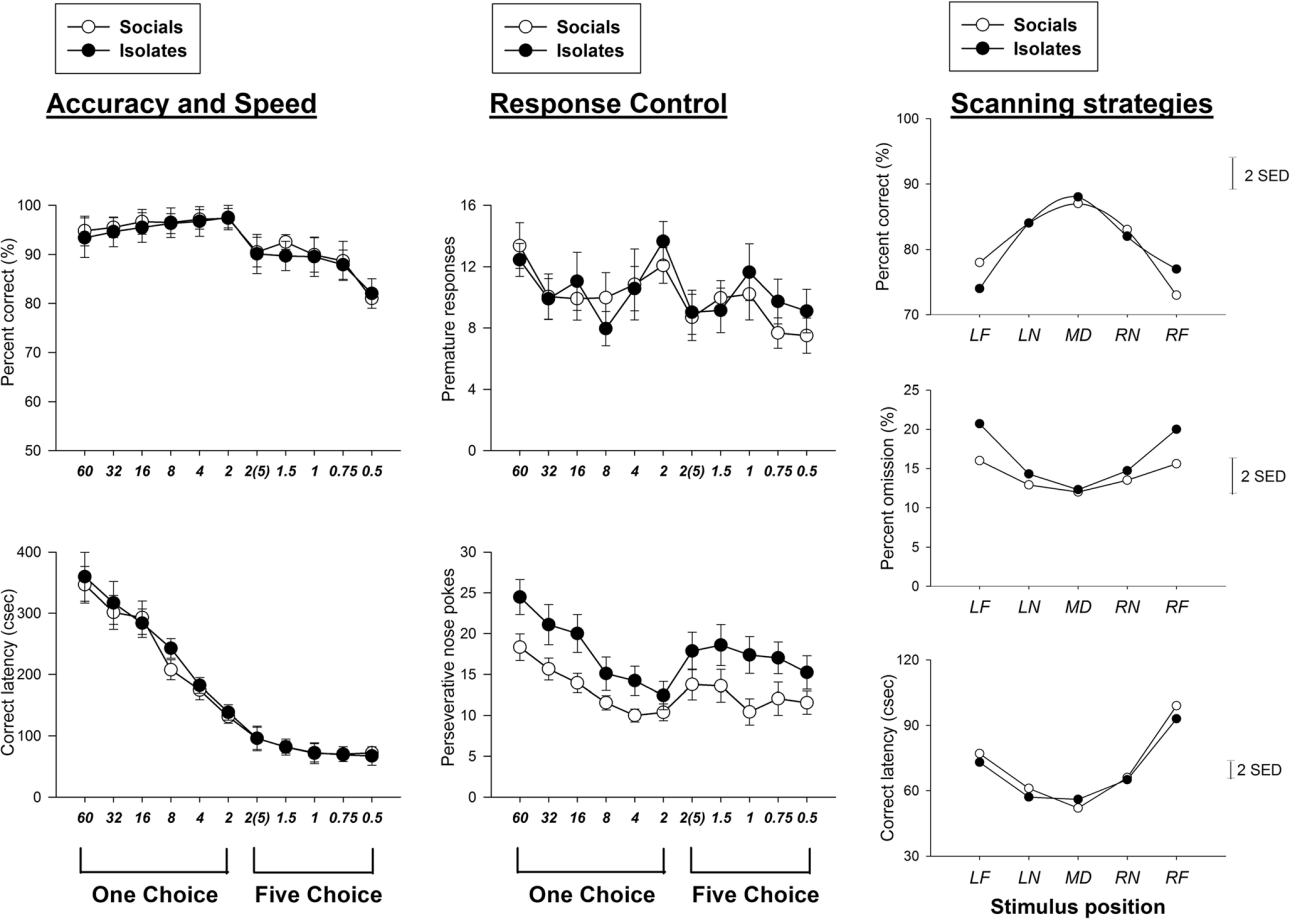
Effects of rearing condition on accuracy and speed, response control and scanning strategies during the acquisition stage of 5-CSRTT. **a** Rearing condition has no effects on percent correct and correct latency (*left panel*). **b** IR rats made more perseverative nose pokes. **c** Both groups adopted similar scanning strategies as scanning from a relative fixed central point of the stimulus array. For *left* and *middle panels*, *values* represent the mean ± SEM. For right panels, and the *vertical bar* represents the standard error for the difference between means (SED) taken from the error terms for the interaction between factors. The relevant formulae are given in Cochran and Cox (1957). As the SED could be used as the denominator for post hoc comparisons with Student’s *t* test, it is an appropriate comparator for the visual evaluation for the difference between two mean values

#### Effects of variable ITIs on 5-CSRTT performance (Fig. 2)

When the ITI was prolonged, both groups exhibited systematic increases in premature responses [*F*(6,132) = 4.32, *p* < 0.001] and decreases in correct latency [*F*(6,132) = 3.92, *p* < 0.001]. There were no effects of rearing condition on accuracy/speed and response control of the 5-CSRTT performance in the variable ITI conditions [percent correct, *F*(1,22) = 0.09; correct latency, *F*(1,22) = 0.27; premature responses, *F*(1,22) = 0.12; and perseverative nose pokes, *F*(1,22) = 0.84; ns for all].

**Fig. 2:**
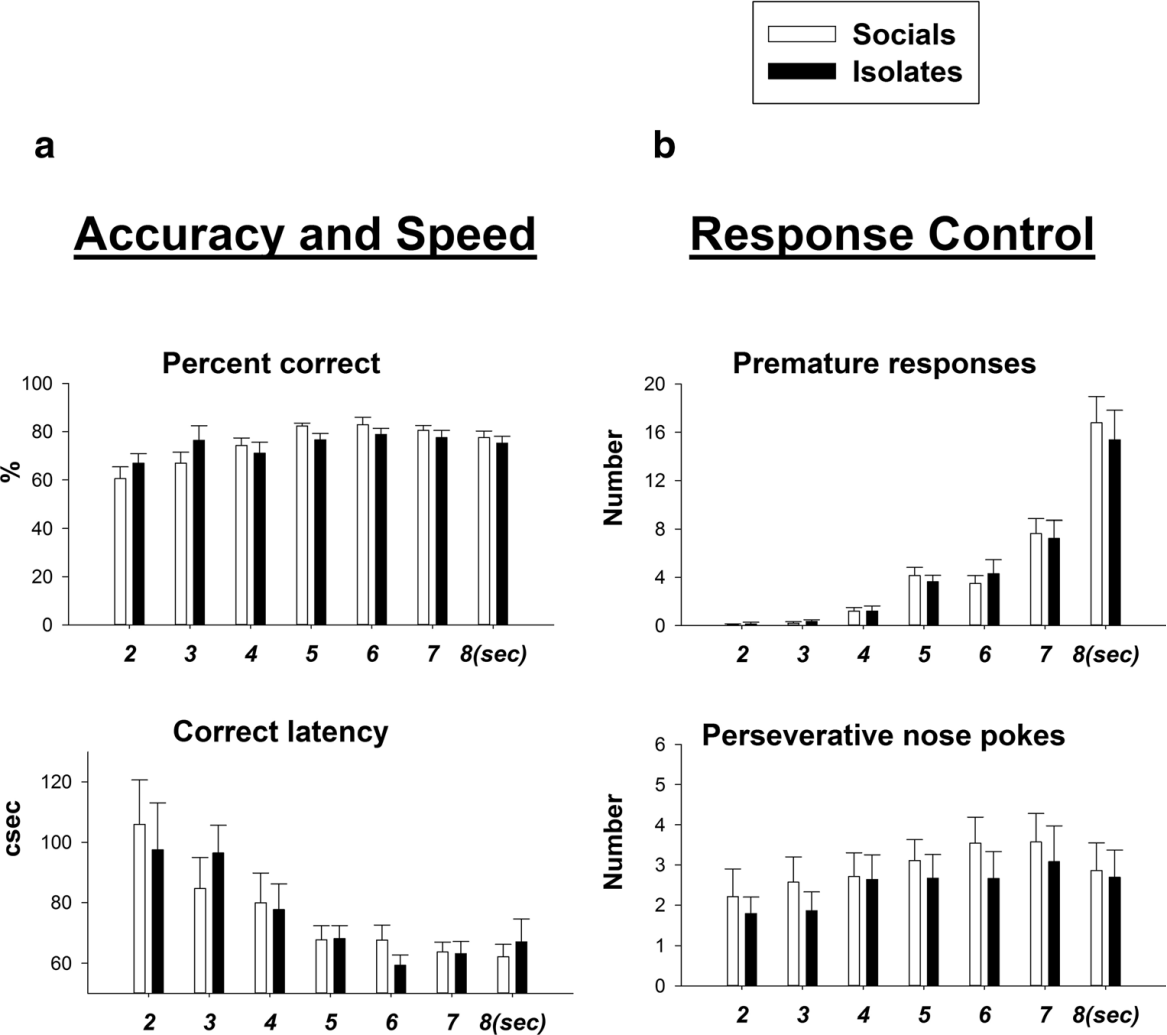
Effects of rearing condition on accuracy/speed and response control when the length of ITI is variable. **a** Rearing condition had no effects on percent correct and correct latency. **b** Rearing condition had no effects on premature responding and perseverative nose poking

#### Effects of rearing condition and amphetamine on 5-CSRTT performance under standard ITI conditions (Fig. 3a)

Amphetamine had no effects on percent correct and correct latency in either rearing condition [all *F*s < 1.0] at the 5-s standard ITI. For premature responding, there was no rearing condition × dose interaction. IR rats exhibited less premature responding than SR rats [main effect of rearing condition, *F*(1,22) = 8.03, *p* < 0.01]. Amphetamine dose-dependently increased premature responding in both groups [main effect of dose, *F*(3,66) = 5.88, *p* < 0.001]. For perseverative responses, there was a dose × rearing condition interaction [*F*(3,66) = 4.25, *p* < 0.01], and IR rats exhibiting fewer perseverative responses than SR rats [main effect of rearing condition, *F*(1,22) = 8.12, *p* < 0.01].

**Fig. 3:**
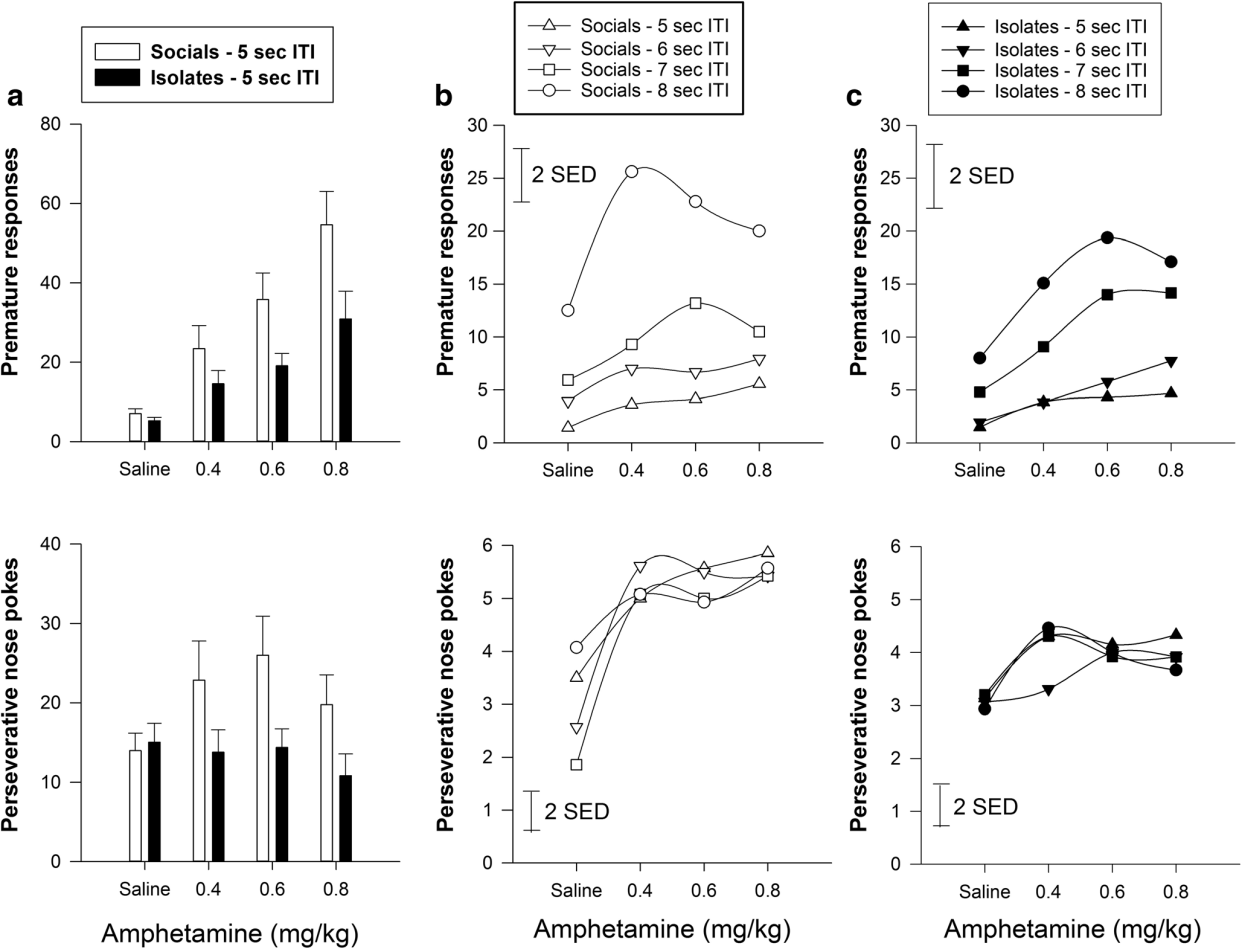
Effects of isolation rearing and amphetamine on response control under both standard testing condition and long variable ITI lengths in 5-CSRTT. **a**. Amphetamine dose-dependently increased premature responding in both groups but IR rats were less impulsive. SR but not IR rats had more perseverative nose pokes under amphetamine challenge. **b** Social controls exhibited a greater magnitude of premature responding at longer ITIs and also a shift of the dose-response curve to the left earlier than isolates. For *middle* and *right panels*, the *vertical bar* represents the standard error for the difference between means (SED) taken from the error terms for the interaction between factors. See *legend* of Fig. 1 for detail

#### Effects of rearing condition and amphetamine on 5-CSRTT performance under conditions of temporal unpredictability (variable, long ITIs) (Fig. 3b, c)

When amphetamine treatment was administered under variations of temporal predictability of the target cues employing long ITIs, three-way ANOVA revealed no significant two- or three-way interactions with rearing, dose and ITI for measures of percent correct, correct latency or perseverative nose pokes (all *F* < 1.0, ns). There were no main effects of rearing condition [percent correct [*F*(1,22) = 1.06; correct latency *F*(1,22) = 0.40; perseverative nose pokes, *F*(1,22) = 3.18, all ns] or dose [percent correct, *F*(3,66) = 0.85; correct latency, *F*(3,66) = 1.19; perseverative nose pokes, *F*(3,66) = 1.39, all ns]. There were only main effects of ITI on percent correct [*F*(3,66) = 5.38, *p* < 0.01] and perseverative responding [*F*(3,66) = 3.82, *p* < 0.05] and a main effect of dose on perseverative responding [*F*(3,66) = 7.35, *p* < 0.01].

The main behavioural effects found were for premature responses, which were increased at those ITIs longer than 5 s and following d-amphetamine (Fig. 3b). Three-way ANOVA revealed significant main effects of ITI [*F*(3,66) = 6.28, *p* < 0.01] and dose [*F*(3,66) = 4.14, *p* < 0.01] but not rearing condition [*F*(1,22) = 0.969, ns]. There were two-way significant interactions for dose x ITI [*F*(9,198) = 2.784, *p* < 0.01] and rearing condition × ITI [*F*(3,66) = 3.803, *p* < 0.05]. The latter indicated that IR rats generally showed less premature responding than SR rats as a function of the ITI, this difference being especially evident at the longest 8-s ITI. Although there was no significant three-way, rearing condition × dose × ITI interaction [*F*(9,198) = 0.598, ns] inspection of the graphs showed differences in effects of amphetamine in the two groups and further post hoc analysis was applied. This showed that SR rats exhibited significantly more premature responding at longer ITIs under amphetamine treatment rather than IR rats [for SR rats, dose × ITI: *F*(9,99) = 2.96, *p* < 0.01; for IR rats, dose × ITI: *F*(9,99) = 0.80, ns]. This difference was particularly apparent at 0.4 mg/kg.

### Experiment 2: impulsive choice

#### TDRT acquisition (Fig. 4)

For simplicity of comparison, the factor session block (four representative stages across the acquisition, sessions 1–4, 9–12, 17–20 and 25–28 were chosen, as they comprised the beginning, the last and the session blocks at equal intervals across the acquisition stage) was used to examine changes in the preference for the larger reward over time. There were no effects of rearing condition on the percentage of choice of the larger reward at each delay block over the training sessions [rearing condition × session block × delay, *F*(12,264) = 0.36, ns]. However, preference for the large reward and tolerance for the delay developed over sessions, as there was a main effect of session block [*F*(3,66) = 12.40, *p* < 0.001] and an interaction between session block and delay [*F*(12,264) = 15.38, *p* < 0.001]. Further analyses were then made separately in four different session blocks. For the percent choice of the larger reward, there were significant main effects of rearing condition in sessions 9–12 [*F*(1,22) = 4.66, *p* < 0.05] and sessions 17–20 [*F*(1,22) = 4.52, *p* < 0.05], and a rearing condition × delay interaction in sessions 25–28 [*F*(4,88) = 2.50, *p* < 0.05]. These results indicate a preference by IR rats for the delayed, larger reward compared to SR rats.

**Fig. 4:**
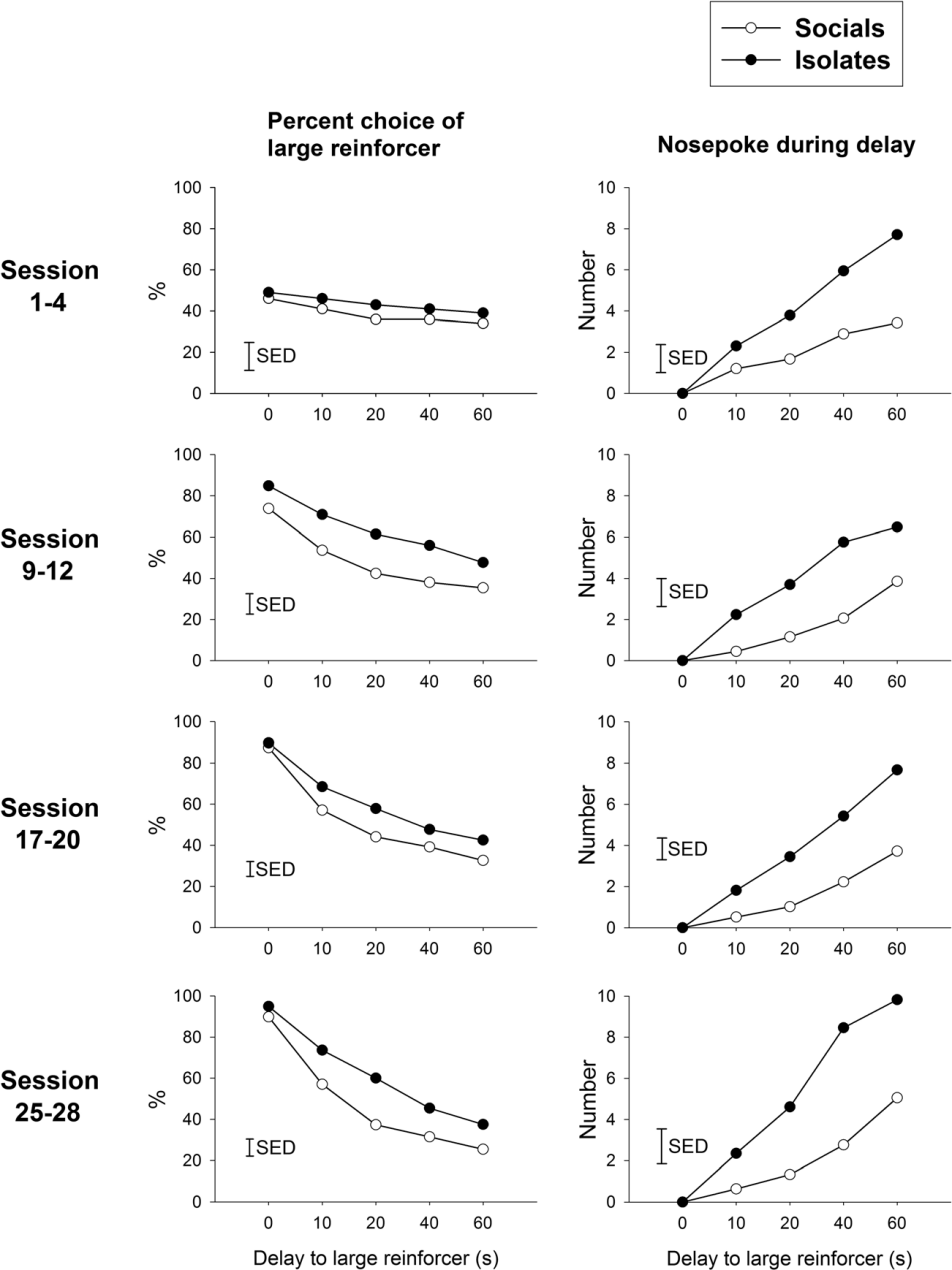
Effects of isolation rearing on TDRT across different acquisition stages on percent choice of large reinforce and nose poking during delay. **a** Both groups successfully developed sensitivity to delay, with isolates exhibiting greater choice for the large but delayed reward. **b** Isolates exhibited higher rates of nose poking while waiting for the large reward pellets. The vertical bar represents the standard error for the difference between means (SED) taken from the error terms for the interaction between factors. See *legend* of Fig. 1 for detail

For nose poking during delay, longer delays increased nose poking across all stages [main effect of delay, *F*(4,88) = 6.90, *p* < 0.001]. This effect was more evident in the IR than the SR rats [main effect of rearing condition, *F*(1,22) = 8.15, *p* < 0.01; interaction of rearing condition × delay, *F*(4,88) = 3.74, *p* < 0.01]. This was a specific effect as it did not occur for nose poking during ITI periods (data not shown).

#### Removal of delay (Fig. 5, upper panel)

When the delay was removed, there was an increase in the preference for the larger reward in all rats [main effect of delay, *F*(1,22) = 9.22, *p* < 0.001] with no influence of rearing condition [*F*(1,22) = 2.54, ns], in which SR [*F*(4,44) = 9.08, *p* < 0.001] and IR rats [*F*(4,44) = 8.96, *p* < 0.001] showed similar sensitivity to the removal of delay across five blocks (i.e. 0, 10, 20, 40 and 60 s). Similarly, there was no effect of the removal of delays on omissions or the latency to collect rewards in either group [all *F*s < 1.0, ns].

**Fig. 5:**
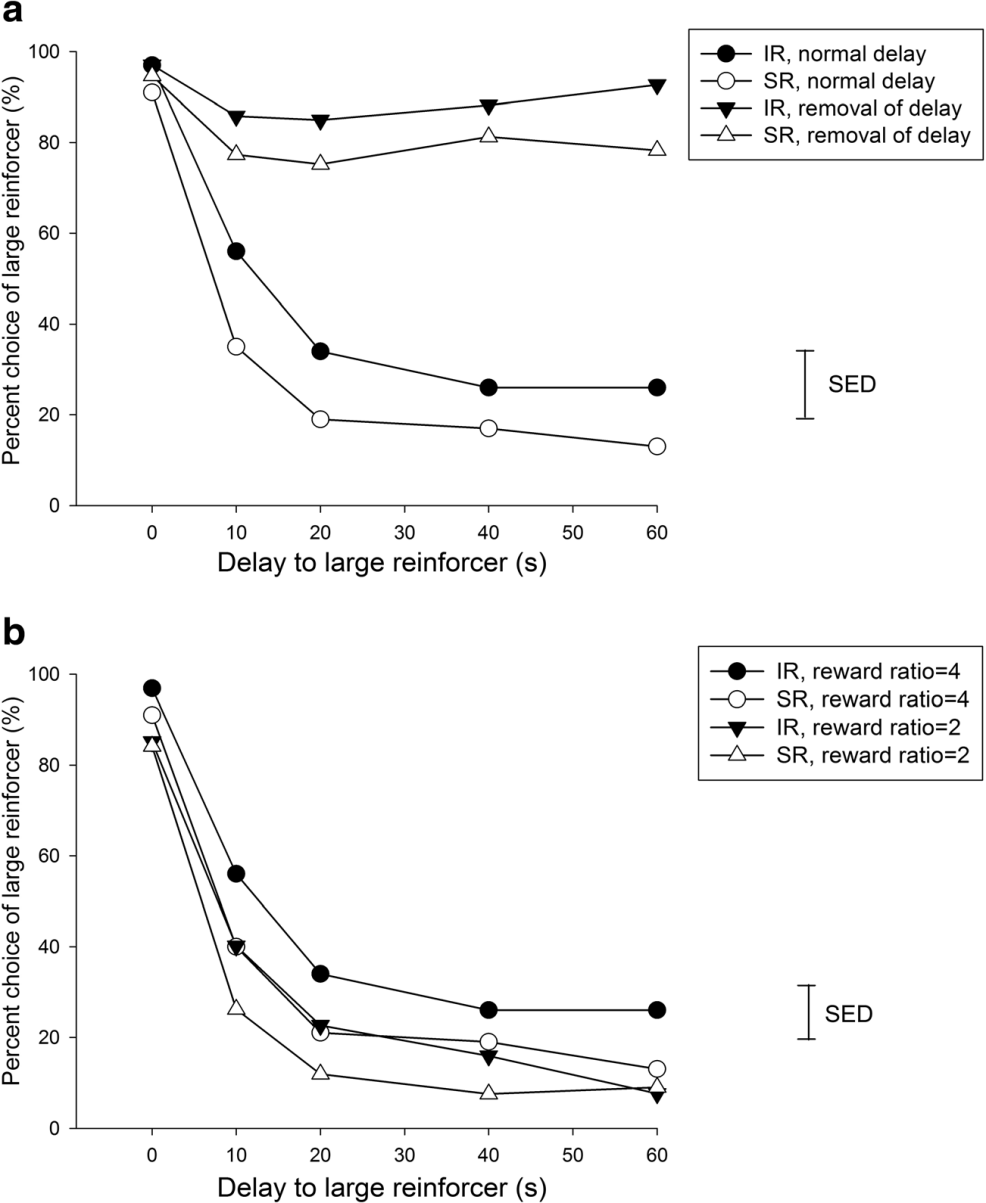
Effects of delay and reward on the performance of isolation rearing on TDRT in socials and isolates. **a** Both groups exhibited a similar sensitivity in choice behaviour in the condition when delay was removed from the task context (**a**). **b** When the reward ratio was reduced, both groups exhibited a choice preference for small, but immediate reward (**b**). The *vertical bar* represents the standard error for the difference between means (SED) taken from the error terms for the interaction between factors. See *legend* of Fig. 1 for detail

#### Change of reward ratio (Fig. 5, lower panel)

For choice behaviour, there was a general reduced preference to wait for the delayed large reward as the amount of reward diminished (main effect of ratio on choice behaviour [*F*(1,22) = 18.26, *p* < 0.001] with no influence of rearing condition [*F*(1,22) = 2.05, ns], also without any interactions with factors rearing condition, ratio and delay). This suggests that in both rearing conditions, the decrease in reward ratio led to an anticipated preference for immediate small reward.

#### Amphetamine effects on TDRT performance (Figs. 6 and 7)

For the percentage of choice of the larger reward, there was a significant effect of dose [*F*(3, 66) = 4.28, *p* < 0.01] independent of the interactions of rearing condition × dose × delay [*F*(12,264) = 1.02, NS] and rearing condition × dose [*F*(3, 66) = 1.48, NS]. Further analyses were made separately to examine the sensitive dose range in terms of rearing condition. For socials, there was a significant effect of dose [*F*(3,33) = 5.12, *p* < 0.01] in which all doses increased the preference for large reward. For isolates, there was a significant effect of dose [*F*(3,33) = 4.76, *p* < 0.01] in which only doses of 0.4 mg/kg [*t*D(4,44) = 3.16, *p* < 0.01] and 0.8 mg/kg [*t*D(4,44) = 2.92, *p* < 0.01] but not 1.5 mg/kg, increased the preference for the large reward.

**Fig. 6:**
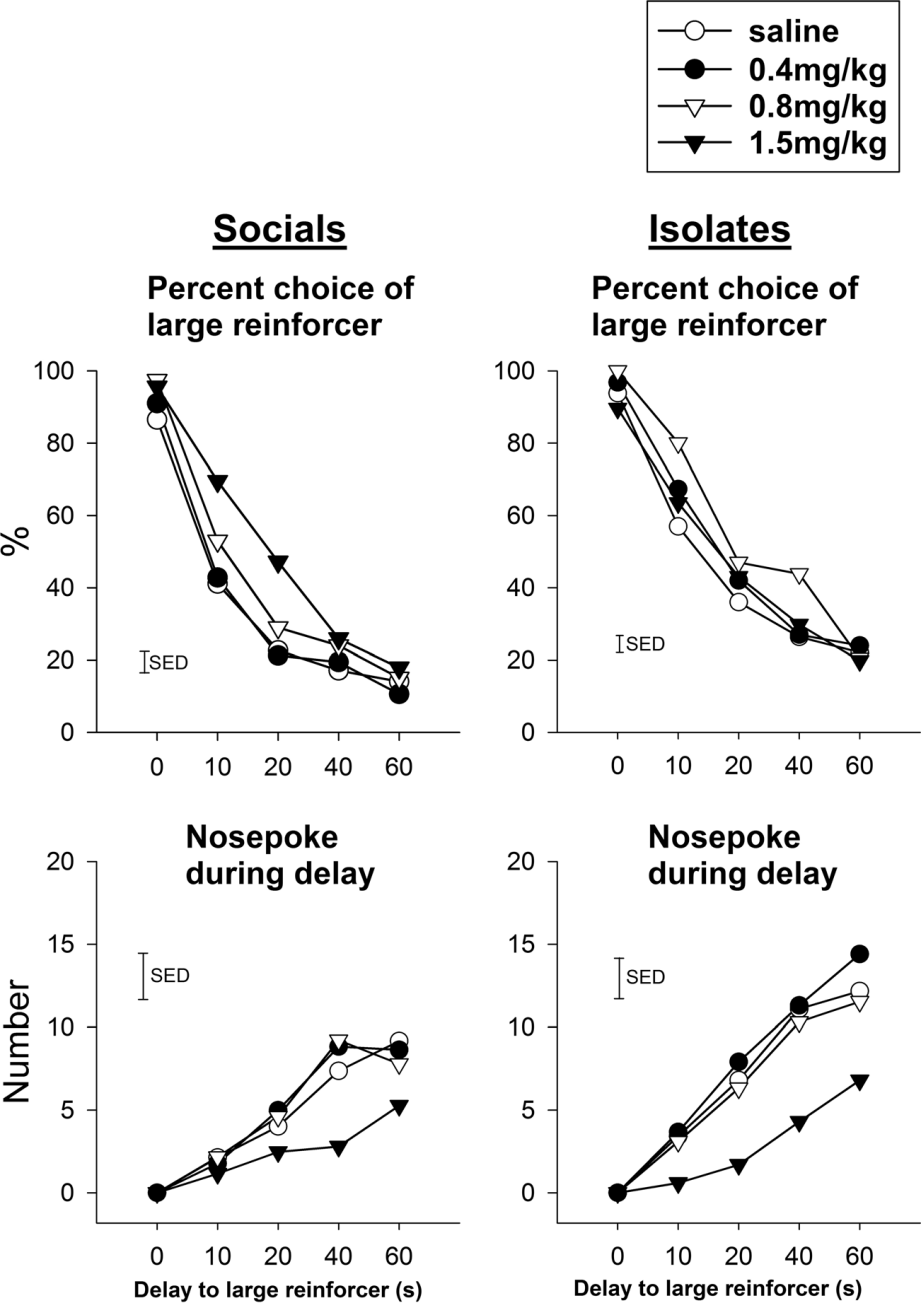
Effects of amphetamine on the performance of TDRT in socials and isolates (intra-group comparisons). **a** Isolates had a compressed sensitivity range than socials to amphetamine in the choice preference to delay but large reward (*upper panels*). **b** Nose poke during delay reductions were more evident in isolates at higher dose of amphetamine (*lower panels*). The *vertical bar* represents the standard error for the difference between means (SED) taken from the error terms for the interaction between factors. See *legend* of Fig. 1 for detail

**Fig. 7:**
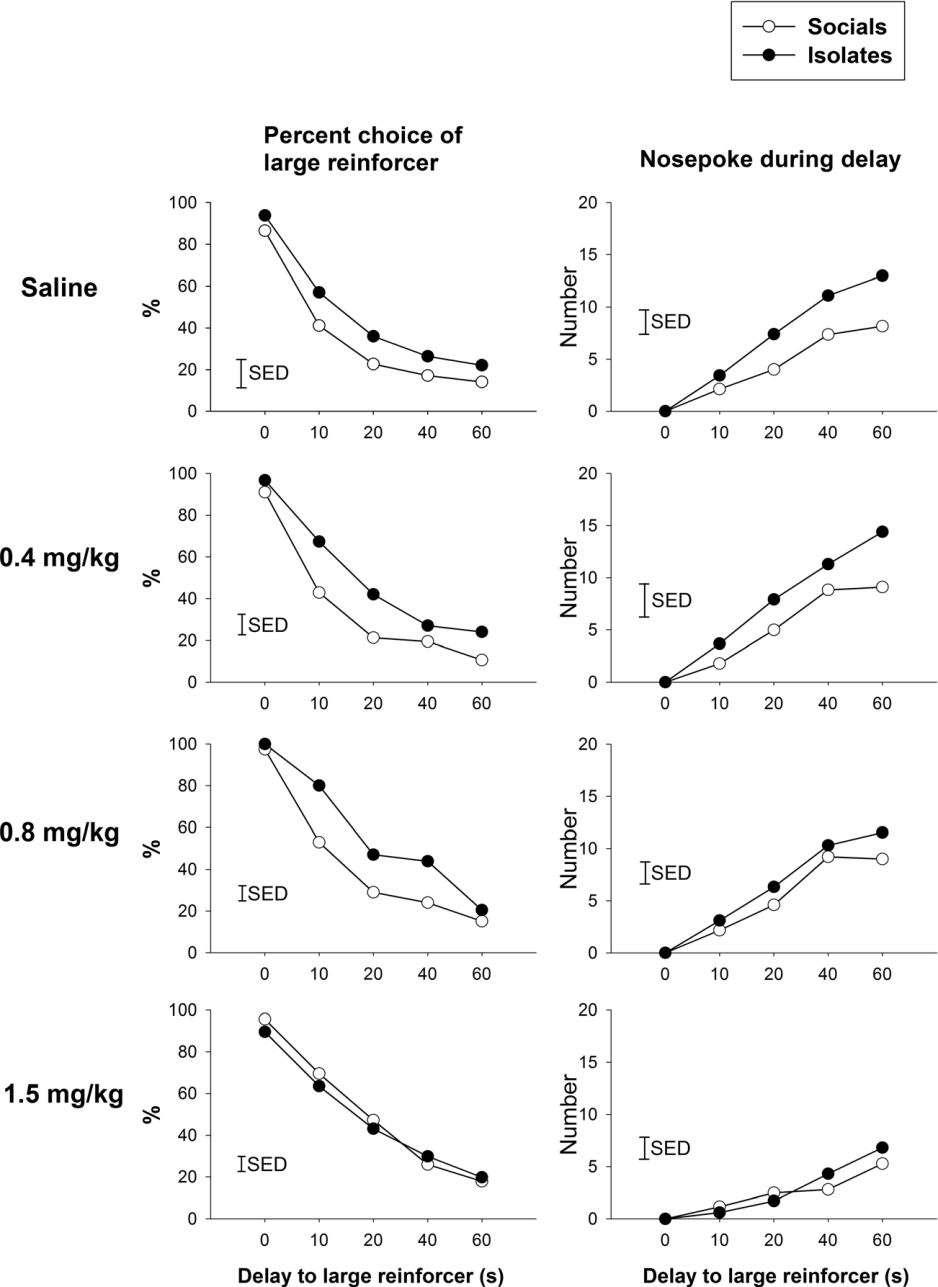
Effects of isolation rearing on TDRT under amphetamine challenge. **a** Isolates please actionexhibit greater choice for the large but delayed reward disappeared at the highest dose of amphetamine (*left panels*). **b**. Higher nose-poking rate during delay in isolates was reduced as the amphetamine dose was increased (*right panels*). The *vertical bar* represents the standard error for the difference between means (SED) taken from the error terms for the interaction between factors. See *legend* of Fig. 1 for detail

For nose poking during delay, there was no significant rearing condition × dose × delay interaction [*F*(12,264) = 1.50, ns]. However, there was a main effect of dose [*F*(3,66) = 9.72, *p* < 0.001] and an interaction between rearing condition × dose [*F*(3,66) = 4.56, *p* < 0.01] in which nose poking was reduced more evidently in isolates at the higher dose of amphetamine. This is in line with the finding that higher nose-poking rate during delay in isolates reduced with increasing dose of amphetamine.

## Discussion

The present study investigated long-term effects of social isolation on two behavioural tasks assessing different types of impulsivity; impulsive action and impulsive choice. Although socially isolated rats were confirmed to be hyperactive, they were nevertheless less impulsive than socially reared animals using two different tests of ‘waiting’ impulsive behaviour, the 5-CSRTT and during acquisition of a test of delayed reward discounting. The IR rats were not impaired in any aspect of 5-CSRTT performance relating to attention. Consequently, it is apparent that IR produces only a small component of the human ADHD syndrome that relating to behavioural hyperactivity only.

In line with Baarendse et al. (2013), our results demonstrated task-dependent, divergent amphetamine effects on motor impulsivity (or impulsive action) and impulsive choice (increasing the former and reducing the latter). Amphetamine treatment helped to reveal differences between the IR and SR groups that were only present transiently or under certain conditions. Thus, IR rats did not show significant reductions in premature responding in the 5-CSRTT under standard testing conditions (5-s ITI) or with variable ITIs, in the absence of the drug. When amphetamine challenges were introduced for both standard test conditions (5-s ITI) and sessions of variable, long ITIs for the 5-CSRTT, the drug increased premature responding in both groups. However, IR rats showed significantly less premature responding overall in the standard testing conditions (5-s ITI). For the long ITI challenge, the results were similar except that at longer ITIs amphetamine proportionately increased premature responding to a greater extent in SR than in IR rats. In the TDRT, IR rats exhibited greater preference for the large but delayed reward during acquisition of the task (i.e. reduced impulsive choice), with a higher rate of nose poking during the delay and a relative reduction in dose-related effects of amphetamine to produce less impulsive choice behaviour during subsequent TDRT performance.

### Response to amphetamine

Previously, IR rats have been shown to be more sensitive to the behavioural effects of d-amphetamine, consistent with a leftwards shift in the dose-response curve (Sahakian et al. 1975; Jones et al. 1990; Wilkinson et al. 1994; Howes et al. 2000), also associated with elevated levels of DA measured using in vivo microdialysis (Jones et al. 1992; Wilkinson et al. 1994). However, in the present study, no leftwards dose-response shift was shown for the drug to increase premature responding on the 5-CSRTT or for impulsive choice on the TDRT. In fact, in both cases, there was a tendency for the IR rats to be mildly *less* sensitive to amphetamine. Although the IR rats had overall reduced premature responding compared with socially reared controls under conditions of both standard testing conditions and variable ITI, they nevertheless generally exhibited increases in responding following amphetamine treatment. However, at long ITIs, this increase was significantly proportionately less than that of controls at 0.4 mg/kg (see Fig. 3, at 8-s ITI). Overall, therefore, there is no major discrepancy with the results of Dalley et al. (2002a) or Zeeb et al. (2013). In general, isolates often exhibit slightly less premature responding at baseline but this difference often becomes significant under challenging conditions (e.g. 8-s ITI or amphetamine treatment). The IR rats are susceptible to rate-increases in premature responses with amphetamine but this effect is sometimes reduced at certain parameters of dose and ITI.

The relative lack of response to amphetamine in the IR rats in the 5-CSRTT is perhaps more noteworthy than may at first be apparent because their often significantly lower premature responding might have been expected to result in a *greater* increase in responding under amphetamine because of the principle of baseline rate-dependency, in which initially low baseline response rates show increases with stimulant drugs, but high rates may show less increase or even a reduction (Robbins 1981). This principle has recently been shown to apply to the effects of the related stimulant drugs methylphenidate and cocaine on premature responding of SR rats in the 5-CSRTT (Caprioli et al. 2015a, 2015b). Thus, individual SR rats with relatively lower baseline levels of premature responding showed *greater* increases with drug treatment than rats with higher baselines (in fact, high impulsive rats actually exhibited significant reductions in premature responding). These effects were also accompanied by similar changes in striatal dopamine D2/3 receptors, which might be relevant to interpreting the effects in the IR rats. IR rats have previously been shown to exhibit increases in high affinity striatal D2 receptors (Jones et al. 1992; King et al. 2009) and enhanced DAT activity (Yorgason et al. 2015)], consistent with a role for striatal D2 receptors in regulating this form of impulsivity. However, striatal and cortical presynaptic DA as well as 5-HT is also probably involved in the regulation of impulsivity in isolates (see Dalley et al. 2002b) and further discussion of these neurochemical substrates is beyond the scope of the present paper.

The reduced sensitivity of the IR rats to amphetamine in the TDRT possibly parallels the narrowed sensitivity of effect across doses of amphetamine in IR rats in a decision-making component of a gambling task (Zeeb et al. 2013). The finding contrasts with the lack of effects shown in a comparable task in Baarendse et al. (2013), probably resulting from the use of only a single dose in that study. It may reflect the rate-dependency principle described above (Robbins 1981). Thus, the reduced dose-related effect of amphetamine on impulsive choice in IR rats might have resulted from the initially greater choice of the large delayed reward in IR rats. These findings also explained the observation that the group difference between SR and IR rats became diminished under the amphetamine challenged condition.

### Effect of isolation rearing on measures of impulsivity

In general, we have been able to show under certain conditions, reduced impulsivity in IR rats in *both* impulsive action and impulsive choice, unlike previous studies which either found increased premature responding under demanding test conditions but with no effect on impulsive choice (Baarendse et al. 2013) or reduced impulsive action, but increased impulsive choice (Hellemans et al. 2005). These two forms of impulsive behaviour have been referred together as ‘waiting impulsivity’ in order to discriminate them from other forms (See Dalley et al. 2011). We conclude that IR can produce a general tendency to reduce waiting impulsive behaviour, despite the hyperactivity evident in these animals. This conclusion is partly at variance with that of Kirkpatrick et al. (2013, 2014) who similarly found evidence of reduced impulsive action, but instead found enhanced impulsive choice produced by IR in their test paradigms, that is, IR rats tended to choose the smaller, sooner option. Possible reasons for the latter discrepancy are that their IR rats were compared with a group of rats reared together in a large social group with additional stimulation provided by environmental enrichment with frequently replaced novel objects. Thus, the key variable may be general environmental enrichment rather than IR per se. Additionally, their IR rats were not handled during the period of isolation, whereas the IR rats in the present study were extensively handled through daily testing.

Intriguingly, Baarendse et al. (2013) found an opposite effect of IR, to increase premature responding in the 5-CSRTT following a period of re-socialization, when the IR rats were tested as adults, although there was no effect on impulsive choice. Presumably, therefore re-socialization, not only reduced the effects of IR to negate potential reductions in motor impulsivity in demanding conditions but also actually produced the reverse effect, namely to increase it. It is thus of considerable interest that IR can apparently lead to opposite effects on motor impulsivity as a joint function of re-socialization and enhanced task demands. We postulate that re-socialization may have precipitated increased impulsive behaviour in the IR rats due to increased stress resulting from social interactions during re-socialization.

Our findings for effects of IR generally to reduce impulsive choice are in agreement with those of Hellemans et al. (2005). Presumably, a period of re-socialization removed this effect in IR rats in the study by Baarendse et al. (2013). These findings can be compared with other effects of re-socialization which probably reflect the diversity of the behavioural and physiological changes caused by IR. Thus IR-induced hyperactivity, but not prepulse inhibition deficits can be normalized by re-socialization (Liu et al. 2011; Wilkinson et al. 1994). Hellemans et al. (2004) also demonstrated that re-socialization reversed certain IR-induced abnormalities (anxiety profiles) but not others (cortical thickness). Moreover, IR-induced startle habituation under REM sleep deprivation can only be achieved after long-term IR (Chang et al. 2014).

### Possible roles of mediating factors in IR syndrome

The low impulsiveness in IR rats could possibly be associated with increases in mediating behaviours. In the TDRT task, IR rats exhibited consistently higher rates of nose poking in the food magazine while waiting for the large reward pellets. (Unfortunately, this behaviour was not monitored in the 5-CSRTT). As this nose poking occurred after the choice had been made, this behaviour could possibly reflect greater incentive motivational effects of the food reward (Jones et al. 1990), as also postulated by Kirkpatrick et al. (2013). However, this interpretation should be made cautiously as previous findings indicate that isolates are slower to collect earned food pellets in the 5-CSRTT (Dalley et al. 2002a).

For the TDRT, two task manipulations in the present study were used to test assumptions of the task (Wogar et al. 1992) with accompanying significant implications for motivational theories of IR. Our results demonstrated that SR and IR rats were equally affected by the removal of delay, similarly shifting to the preference of choosing the shorter delay reward when a lower reward ratio was introduced, in line with the findings of Wogar et al. (1992) and the expectation that rats should become more impulsive in this task when reinforcer value was reduced. These data also support the conclusion that IR rats do not show alterations in values of reinforcers based on the hyperbolic model of impulsive choice (Brunner and Hen 1997; Stein et al. 2013). Note that a similar manipulation by Kirkpatrick et al. (2013) suggested greater sensitivity to reinforcement by their IR rats which led them to postulate their effects on impulsive choice depended on this factor.

Alternatively, the increase in mediating behaviours could be regarded as an ‘adjunctive’ form of behaviour to cope with, or relieve, hypothetically heightened anxiety experienced by the rats while waiting, as also observed in schedule-induced polydipsia (Brett and Levine 1979, 1981). However, this interpretation of effects of IR also appears unlikely, as a previous study has shown that IR rats are not in general more prone to exhibit more adjunctive behaviours. Jones et al. (1989) showed that isolation rearing *retarded* the acquisition of schedule-induced polydipsia, possibly because it less effectively reduced corticosterone levels in IR rats. Therefore, whether greater interference or response competition by other mediating behaviours can explain the reduced impulsivity in IR rats in either test paradigms is unclear; the origin of the elevated mediating behaviour also remains to be established.

## Conclusions

In summary, our results demonstrate that, despite their hyperactivity in photocell cages, IR rats were generally less impulsive under certain conditions, both on the 5-CSRTT (impulsive action) and on the TDRT (impulsive choice) than their socially reared counterparts. In other words, IR rats exhibited enhanced impulse control over, not only the ‘preparation and execution stage’ (as seen in the 5-CSRTT) but also the ‘outcome evaluation stage’ of behaviour (as seen in the TDRT) (Evenden 1999a, 1999b). This result supports the hypothesis that ADHD symptoms, such as hyperactivity, inattention and impulsivity can be dissociated (Sjöwall et al. 2013). Moreover, IR appears not to be appropriate for modelling the impulsivity domain of ADHD, although it is a means for more generally for investigating effects of early social adversity on brain and behaviour. IR rats were also found to be both *less* sensitive to the usual effects of amphetamine to enhance impulsive behaviour in the 5-CSRTT and also *less* sensitive to the contrasting effect of the drug to reduce impulsive choice in the TDRT, results which have implications for previous findings for IR in other test paradigms, and for understanding the neurochemical substrates of isolation rearing.

## Electronic supplementary material

Supplementary Figure 1 (JPEG 2336 kb).
