# Supplementary figures and images for: ‘Waiting impulsivity’ in isolation-reared and socially-reared rats: effects of amphetamine
Source: Psychopharmacology (Berl). 2017 Mar 17;234(9):1587–601. doi: 10.1007/s00213-017-4579-8 (PMC5420383; doi:10.1007/s00213-017-4579-8)

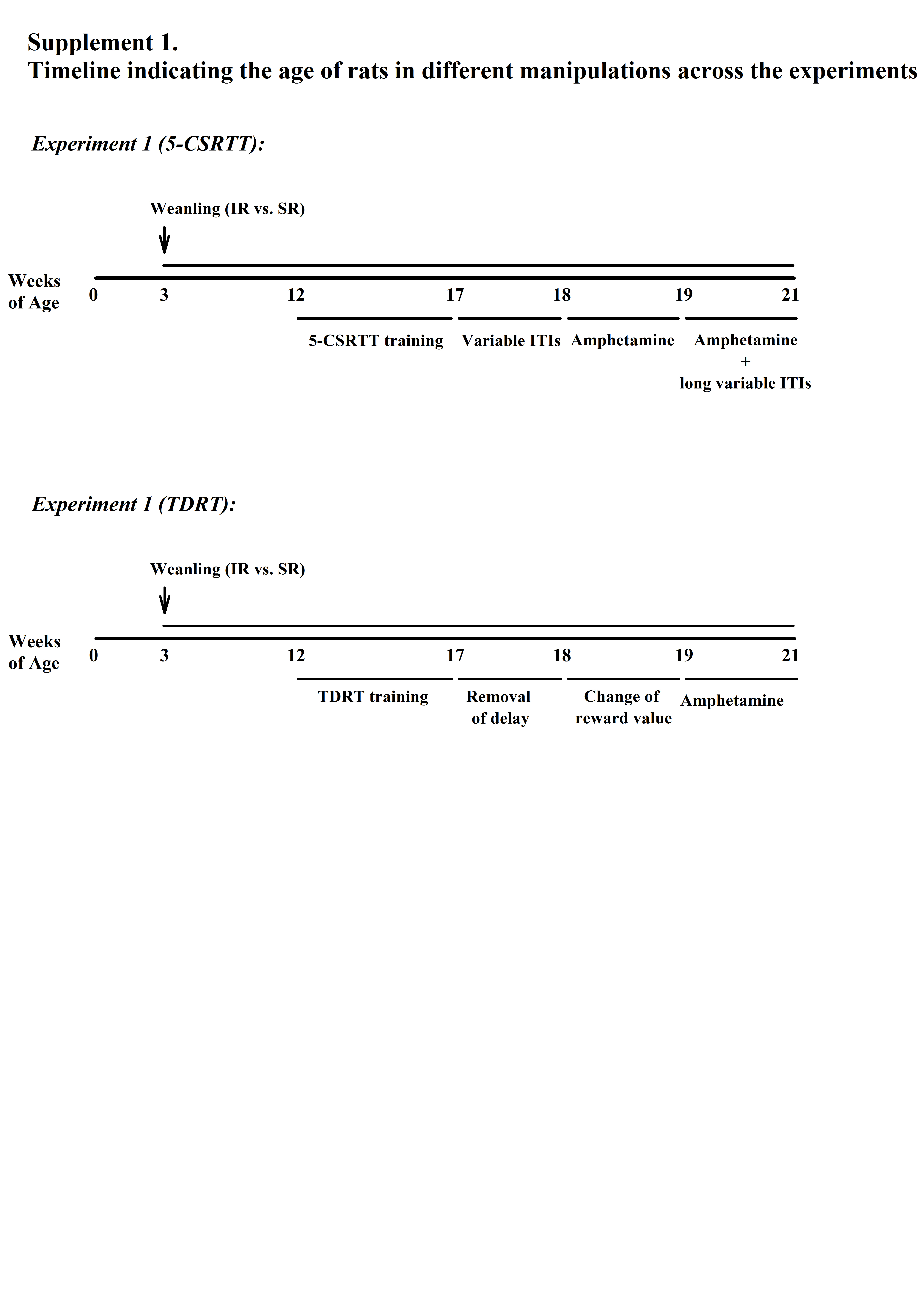

Supplement: Supplementary file 1 — (JPEG 2336 kb). [file 213_2017_4579_Fig8_ESM.jpg]
